# Redundancy, Feedback, and Robustness in the *Arabidopsis thaliana BZR/BEH* Gene Family

**DOI:** 10.3389/fgene.2018.00523

**Published:** 2018-11-13

**Authors:** Jennifer Lachowiec, G. Alex Mason, Karla Schultz, Christine Queitsch

**Affiliations:** ^1^Department of Genome Sciences, University of Washington, Seattle, WA, United States; ^2^Molecular and Cellular Biology Program, University of Washington, Seattle, WA, United States

**Keywords:** developmental robustness, stochasticity, canalization, plant, hypocotyl, variance, BES1, BZR1

## Abstract

Organismal development is remarkably robust, tolerating stochastic errors to produce consistent, so-called canalized adult phenotypes. The mechanistic underpinnings of developmental robustness are poorly understood, but recent studies implicate certain features of genetic networks such as functional redundancy, connectivity, and feedback. Here, we examine the *BZR*/*BEH* gene family, whose function contributes to embryonic stem development in the plant *Arabidopsis thaliana*, to test current assumptions on functional redundancy and trait robustness. Our analyses of *BZR*/*BEH* gene mutants and mutant combinations revealed that functional redundancy among these gene family members is not necessary for trait robustness. Connectivity is another commonly cited determinant of robustness; however, we found no correlation between connectivity among gene family members or their connectivity with other transcription factors and effects on developmental robustness. Instead, our data suggest that *BEH4*, the earliest diverged family member, modulates developmental robustness. We present evidence indicating that regulatory cross-talk among gene family members is integrated by *BEH4* to promote wild-type levels of developmental robustness. Further, the chaperone HSP90, a known determinant of developmental robustness, appears to act via BEH4 in maintaining robustness of embryonic stem length. In summary, we demonstrate that even among closely related transcription factors, trait robustness can arise through the activity of a single gene family member, challenging common assumptions about the molecular underpinnings of robustness.

## Introduction

Development relies on the coordinated action of low concentrations of regulatory factors diffusing within and between cells, which inevitably results in random developmental errors. Typically, organisms tolerate developmental errors, resulting in canalized, wild-type-like individuals (Waddington, 1942; Masel and Siegal, 2009; Lempe et al., 2012; Whitacre, 2012; Félix and Barkoulas, 2015). Robustness to developmental errors is an intrinsic property of all organisms and is genetically controlled (Hall et al., 2007; Ansel et al., 2008; Sangster et al., 2008b; Jimenez-Gomez et al., 2011; Rinott et al., 2011; Ayroles et al., 2015; Metzger et al., 2015; Gonzalez et al., 2016; Pieper et al., 2016; Katsanos et al., 2017). However, the molecular mechanisms that regulate developmental robustness are poorly understood, which is largely due to the technical obstacles of studying this phenomenon in complex, multicellular organisms.

Regulation of developmental robustness has been attributed to a handful of molecular mechanisms and features of gene regulatory networks (reviewed in Masel and Siegal, 2009; Lempe et al., 2012; Whitacre, 2012; Félix and Barkoulas, 2015; Lachowiec et al., 2015b; Hallgrimsson et al., 2018). In *Caenorhabditis elegans*, large-scale double mutant analysis identified several highly connected chromatin modifiers as positive regulators of developmental robustness (Lehner et al., 2006). In *Arabidopsis thaliana*, QTL mapping for regulators of developmental robustness found evidence that the pleiotropic genes *ERECTA* and *ELF3* regulate developmental robustness (Hall et al., 2007; Jimenez-Gomez et al., 2011); both genes are also highly connected in genetic networks. Nevertheless, these and other plant studies suggest that robustness modulators act in a single or limited number of traits rather than in a global manner, presumably through epistasis with specific partner genes.

The protein chaperone HSP90, known for its role in promoting genetic robustness (Rutherford and Lindquist, 1998; Queitsch et al., 2002; Yeyati et al., 2007; Jarosz and Lindquist, 2010; Lachowiec et al., 2013, 2015a; Rohner et al., 2013), also maintains developmental robustness. For example, HSP90 perturbation across many isogenic plants results in vastly increased phenotypic variation (Queitsch et al., 2002; Sangster et al., 2007, 2008b). Similarly, naturally low levels of HSP90 correlate with greater penetrance of mutations in isogenic nematodes (Burga et al., 2011; Casanueva et al., 2012). HSP90’s apparently global role in developmental robustness of plants and animals is consistent with the chaperone’s exceedingly high connectivity in genetic networks (i.e., epistasis with many different partner genes), particularly with many genes encoding kinases and transcription factors important for growth and development (Taipale et al., 2010; Lachowiec et al., 2015a).

Theoretical and empirical studies suggest that developmental robustness emerges from the circuitry of genetic networks. For example, highly connected nodes in genetic networks may be of particular importance in regulating robustness to noise due to their many interactions (Lehner et al., 2006; Levy and Siegal, 2008; Masel and Siegal, 2009; Whitacre, 2012). Another feature of genetic networks commonly associated with developmental robustness is functional redundancy among genes (Gutiérrez and Maere, 2014). Functional redundancy will compensate for stochastic losses of function in specific gene family members or paralogs (DeLuna et al., 2008, 2010).

Gene duplication is one obvious source of functional redundancy, and thereby developmental robustness. In *A. thaliana*, one-third of genes belong to multi-member gene families (Swarbreck et al., 2008), which have arisen through three well-supported whole genome duplications (Simillion et al., 2002; Bowers et al., 2003), in addition to segmental and tandem duplication events (The Arabidopsis Initiative, 2000). Duplication of transcription factor genes provides a plausible but potentially complex form of robustness regulation. Transcription factor family members recognize highly similar DNA motifs (Franco-Zorrilla et al., 2014), and often regulate one another (Phillips and Hoopes, 2008), showing functional redundancy as well as feedback regulation (Wang et al., 2012; Sullivan et al., 2014; Lachowiec et al., 2015b). At the same time, transcription factors are particularly vulnerable nodes for developmental robustness due to their often low cellular concentrations and positions as both master regulators (Chan and Kyba, 2013) and convergence points of signaling cascades (Li et al., 2014). It is unclear how these different features of transcription factors and their gene families converge to regulate developmental robustness.

The *BES1/BZR1 HOMOLOG* (*BEH)* transcription factors belong to a small gene family exclusive to plants. With only six members (Wang et al., 2002), this family is tractable for studying the role of redundancy, connectivity, and feedback on developmental robustness. The well-studied founding members of the *BZR/BEH* family, *BRI1-EMS-SUPRESSOR1* (*BES1*) and *BRASSINAZOLE-RESISTANT1* (*BZR1*) result from the most recent whole genome duplication in the *A. thaliana* lineage and are highly similar in sequence (Blanc et al., 2003). They are thought to be the primary transcription factors in brassinosteroid signaling, and studies of their phenotypic effects are largely restricted to dominant mutants (Wang et al., 2002; Yin et al., 2002; Zhao et al., 2002). Brassinosteroid signaling regulates a large number of physiological processes in plants, ranging from seed maturation to senescence (Clouse, 2002). Brassinosteroids are recognized by the membrane-associated receptor BRI1 that then represses the activity of the GSK3 kinase BIN2. In the absence of brassinosteroids, BIN2 phosphorylates and inhibits BES1 and BZR1 (Zhao et al., 2002). In this phosphorylated state, BES1 and BZR1 are prohibited from entering the nucleus (Gampala et al., 2007). In the presence of brassinosteroids, BES1 and BZR1 are dephosphorylated (Tang et al., 2011) and localize to the nucleus, where they activate and repress different sets of targets genes (He et al., 2005; Yin et al., 2005; Sun et al., 2010; Yu et al., 2011). BES1 and BZR1 are known to interact with several other proteins to regulate transcription. For example, BES1 dimerizes with BIM family proteins (Yin et al., 2005) to increase DNA binding affinity *in vitro*, interacts with its target gene MYBL2 (Ye et al., 2012), and works with ISW1 (Li L. et al., 2010), ELF6, and REF6 (Yu et al., 2008) to alter chromatin accessibility. Some studies have revealed differences in BES1 and BZR1 protein interactions. For example, BES1, but not BZR1, interacts with the known robustness regulator HSP90 (Lachowiec et al., 2013; Shigeta et al., 2013).

In contrast, the other family members *BEH1-4* are little studied, largely due to the lack of well-characterized loss-of function or dominant mutants. Just as BES1 and BZR1, BEH1-4 are thought to act as transcription factors (Wang et al., 2002; He et al., 2005). Moreover, BEH1, BEH2, BEH3, and BEH4 are phosphorylated in a manner similar to BES1 and BZR1 (Yin et al., 2005), and yeast two-hybrid analyses show that BEH2, in addition to BES1 and BZR1, interacts with a GSK3 kinase (Rozhon et al., 2010). In sum, previous studies support that BEH1-4 could act redundantly with the well-studied transcription factors BES1 and BZR1 (Krizek, 2009; Ye et al., 2012).

Here, we examined the *BZR/BEH* family for effects on developmental robustness through the lenses of redundancy, connectivity, and feedback. Contrary to commonly held assumptions about the importance of redundancy and connectivity in robustness, we trace robustness in hypocotyl growth to the action of a single gene, *BEH4*, which appears to maintain proper cross-talk among *BZR/BEH* family members. Further, we trace HSP90’s role in maintaining robustness of hypocotyl length to the function of *BEH4*, thereby elucidating how this well-known regulator of global developmental robustness may affect this specific trait.

## Results

### *BZR/BEH* Family Members Share Function in Regulating Hypocotyl Elongation in the Dark

To compare the individual functions of different members of a gene family, equivalent mutants facilitate genetic analysis. For studies of *BES1* and *BZR1*, researchers have largely relied on the dominant mutants *bes1-D* and *bzr1-1D*, which introduce the same nucleotide change in their respective PEST domains (Wang et al., 2002; Yin et al., 2002). This mutation appears to stabilize PEST interaction with a phosphatase PP2A (Tang et al., 2011), thereby creating dominant mutants that are constitutively active. Not all members of the *BZR/BEH* family are predicted to contain homologous PEST domains (Rogers et al., 1986) (Supplementary Figure S1), so comparable dominant mutants cannot be created. To assay comparable mutants, we acquired T-DNA insertion mutants for each gene family member (*bes1-2, bzr1-2, beh1-1, beh2-1, beh3-1*, and *beh4-1*, see section “Materials and Methods,” Supplementary Figure S1) (Lamesch et al., 2012). Using qPCR analysis, we assayed each mutant for expression of the respective gene. For *bes1-2, beh1-1*, and *beh3-1* no expression was detected. For *bzr1-2, beh2-1* and *beh4-1* expression was detected for short (< 180 bp) qPCR products, but no full-length or spliced transcript was detected (Sandhu et al., 2013). With these mutants, we performed phenotypic comparisons, as described below.

**FIGURE 1:**
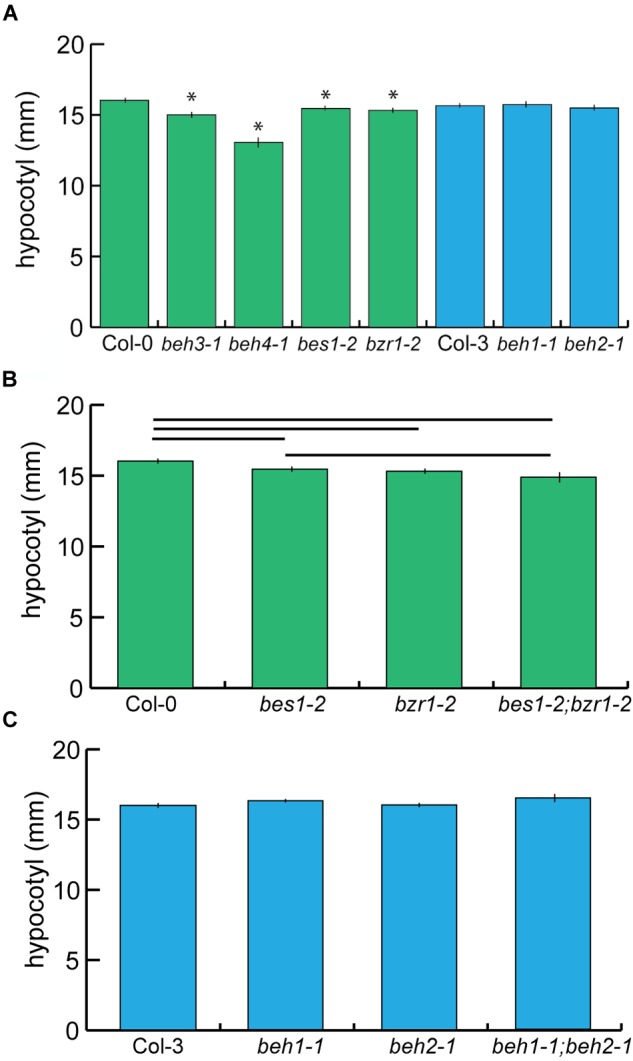
The *BZR/BEH* family encodes genes with similar effects on hypocotyl length. **(A)** Seedlings were grown for seven days in the dark, and hypocotyls were measured. *beh3-1, beh4-1, bes1-2, bzr1-2* hypocotyls were significantly shorter than those of wild-type (^∗^*p* < 0.0001, linear mixed effects model with genotype as a fixed effect and replicate as a random effect). **(B)** The phenotype of the *bes1-2;bzr1-2* double mutant suggests that *BZR1* is epistatic to *BES1* because there was no significant difference in hypocotyl length between *bes1-2* and *bes1-2;bzr1-2*. Significant differences (*p* < 0.05) are displayed by the horizontal bars as determined by linear mixed effect modeling. **(C)** No significant differences in hypocotyl length were observed for *beh1-1* and *beh2-1* single mutants, or for the double mutant *beh1-1;beh2-1*. For (**A–C)** one representative replicate experiment with standard error of the mean for *n* > 20 is shown.

The phenotypes of *bes1-D* and *bzr1-1D* included hyper-elongation of hypocotyls when grown in the dark (Wang et al., 2002; Yin et al., 2002), suggesting that BEH1, BEH2, BEH3, and BEH4 may also promote hypocotyl growth. Indeed, the *bes1-2, bzr1-2, beh3-1*, and *beh4-1* mutants produced significantly shorter hypocotyls than wildtype in the dark (Figure 1A, *p* < 0.0001, linear mixed effects model, *n* = 70). Our results are consistent with previous findings in which RNAi targeting of *BES1* reduces hypocotyl length (Yin et al., 2005; Wang et al., 2013), and the *bes1-1* T-DNA insertion mutant exhibits reduced hypocotyl length (He et al., 2005). Curiously, the mutants of the founding and best-studied members of the *BZR/BEH* family, *BES1* and *BZR1*, were not the most affected in dark growth; rather the mutants of the earliest diverging members *BEH3* and *BEH4* showed larger effects on dark growth, with *beh4-1* exhibiting the strongest defect (Figure 1A). The small but significant effects in these four mutants indicate that these gene family members share function but are not fully redundant in regulation of hypocotyl growth in the dark.

There was no significant difference in dark growth between *bes1-2* and *bzr1*-*2* mutant seedlings, suggesting that *BES1* and *BZR1* contribute to dark growth to the same degree (Figure 1A). This finding is consistent with the similar phenotypes of the dominant *bes1-D* and *bzr1-1D* mutants (Lachowiec et al., 2013); it is also consistent with the high sequence identity between *BES1* and *BZR1* (Wang et al., 2002) and their overlapping patterns of expression (Wang et al., 2002; Yin et al., 2002). To explore whether BES1 and BZR1 independently (i.e., additively) regulate dark growth, we examined the *bes1-2;bzr1-2* double mutant. The double mutant tended to be shorter than either single mutant but was only significantly shorter than *bes1-2* (Figure 1B), suggesting that *BZR1* is epistatic to *BES1* in promoting hypocotyl growth in the dark. Thus, although *BZR1* and *BES1* do not act fully redundantly in hypocotyl elongation, they appear to have overlapping rather than independent functions in its regulation. We speculate that these degenerate functions of *BES1* and *BZR1* may arise from different interacting protein partners. Notably, the *beh4-1* single mutant was significantly shorter than the *bes1-2;bzr1-2* double mutant (*p* < 0.0001, linear mixed effects model, *n* = 70), suggesting a central role of *BEH4* in controlling dark growth.

Among the six family members, we did not detect effects of *beh1-1* and *beh2-1* on dark growth in our assays relative to their respective reference background Col-3 (Figure 1A). Both genes are similar in amino acid sequence (55%). To explore potential functional redundancy between *BEH1* and *BEH2*, we created the respective double mutant and assessed hypocotyl growth. The double mutant *beh1-1;beh2-1* exhibited no significant growth defect compared to wild-type, or the single mutants *beh1-1*, or *beh2-1* (Figure 1C). This result indicates either that *BEH1* and *BEH2* do not regulate hypocotyl elongation or that they act redundantly with other family members or other, unrelated genes in regulating dark growth. Taken together, at least BES1, BZR1, BEH3, and BEH4 share the function of regulating hypocotyl growth during growth in the dark.

In addition to skotomorphogenesis, *BES1* and *BZR1* are also important for photomorphogenesis and flowering (Li J. et al., 2010) based on *bes1-D* and *bzr1-1D* phenotypes. BES1 is known to interact with the flowering-time regulating proteins, ELF6 and REF6 (Yu et al., 2008). We detected no significant defects for the *BZR/BEH* family mutants for flowering time (Supplementary Figure S2), which agrees with earlier findings for a *BES1* T-DNA insertion line, *bes1-1* (He et al., 2005).

When grown in the light, *bes1-D* and *bzr1-1D* exhibit opposing effects on hypocotyl growth, with *bzr1-1D* showing shortened hypocotyls (He et al., 2005; Gampala et al., 2007). In previous work, *bes1-1* showed reduced growth in the light (He et al., 2005). Therefore, we examined all family mutants for light growth. As light-grown seedlings have very short hypocotyls, at least 70 seedlings per genotype were required to detect significant differences for an effect size of 0.5 mm (power analysis, power = 0.8). We hypothesized that the *bzr1-2* would show longer hypocotyls than wild-type in the light, based on the shortened *bzr1-1D* phenotype. Indeed, *bzr1-2* showed significantly longer hypocotyls than wild-type (*p* = 0.0087, linear mixed effects model, *n* = 70, Supplementary Figure S3). In summary, our results reveal partial functional redundancy among these closely related transcription factors.

### *BEH4* Function Maintains Robustness

**FIGURE 2:**
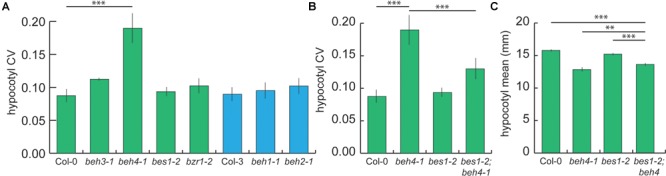
*beh4-1* decreases robustness of dark grown hypocotyls | **(A)** The *beh4-1* mutant exhibits significantly greater variation in hypocotyl length compared to wild-type (^∗∗∗^*p* < 0.0001, Levene’s test, *n* = 210). None of the other single mutants increase hypocotyl length variance significantly. **(B)** The double mutant *bes1-2;beh4-1* showed an intermediate effect on hypocotyl length robustness compared to either single mutant (^∗∗∗^*p* < 0.0001, Levene’s test, *n* = 210). CV was estimated in three biological replicates. Standard error of the mean for *n* = 3 is shown for both **(A)** and **(B)**. **(C)** The double mutant *bes1-2;beh4-1* also showed an intermediate effect on hypocotyl mean values compared to either single mutant (^∗∗∗^*p* < 0.0001, ^∗∗^*p* < 0.001, linear mixed effects model with genotype as a fixed effect and replicate as a random effect).

We hypothesized that the observed similar functions of BES1, BZR1, BEH3, and BEH4 may contribute to developmental robustness of dark grown hypocotyls (Wagner, 2000; Gu et al., 2003; Lachowiec et al., 2015b). Measuring developmental robustness is straightforward in isogenic lines. By growing isogenic lines randomized in the same controlled environment, any variation in phenotype is attributed to errors in development and used as a measure of developmental robustness (Waddington, 1942; Queitsch et al., 2002). Developmental robustness is often expressed as the coefficient of variation or CV (S^2^/Ȳ) (Lempe et al., 2012; Geiler-Samerotte et al., 2013; Gutiérrez and Maere, 2014). We measured hypocotyl length with high replication in the *BZR/BEH* family single mutants using a randomized design to control for micro-environmental differences. Mutants in the founding members of the *BZR/BEH* family, *bes1-2* and *bzr1-2* did not significantly affect developmental robustness in hypocotyl length. Rather, *beh4-1* showed a highly replicable and significant decrease in developmental robustness (Figure 2A, p = 3.145 × 10^-7^, Levene’s test, *n* = 210). No other single mutant significantly affected robustness. Notably, no differences in developmental robustness were observed in light-growth hypocotyls (Supplementary Figure S3). We conclude that robustness in dark grown hypocotyls was most affected by *beh4-1*, which also affected trait mean the most (Figure 1A). This result recalls the results of a prior study, in which we found that HSP90-dependent loci for developmental robustness of dark grown hypocotyls often coincide with those for trait means upon HSP90 perturbation (Sangster et al., 2008b).

We hypothesized that we may observe a further loss of robustness by introducing an additional *BZR/BEH* family mutation. We examined the *bes1-2;beh4-1* double mutant because both single mutants affected mean hypocotyl length in the dark. Surprisingly, we found that introducing *bes1-2* activity partially rescued developmental robustness in the double mutant (Figure 2B). Similarly, trait mean was rescued in *bes1-2;beh4-1* compared to the single *beh4-1* mutant (Figure 2C). We conclude that *bes1-2* and *beh4-1* do not have the same effect on hypocotyl developmental robustness and trait means. Rather, we suggest that developmental robustness arises through the integrated activity of at least BES1 and BEH4. Note that *bes1-2* alone did not affect developmental robustness. It is only through its interaction with *BEH4* that we observed its apparently stabilizing effect. Indeed, others have demonstrated that BES1 directly or indirectly regulates *BEH4* as shown by ChIP-analysis, at least in aerial tissues (Yu et al., 2011).

### Expression Feedback Among Members of the *BZR/BEH* Family in the Light and Dark

**FIGURE 3:**
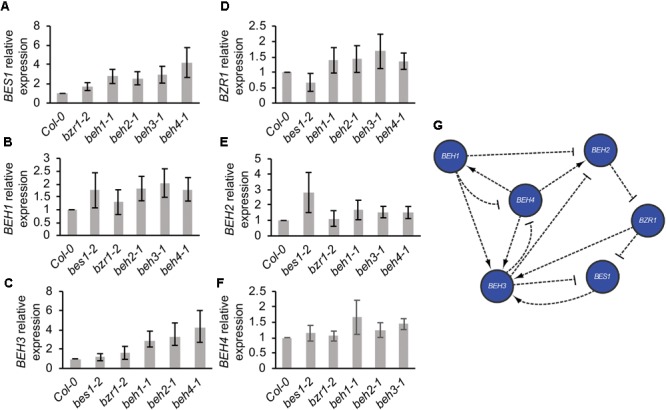
*BZR/BEH* family members engage in extensive regulatory cross-talk. Level of gene expression (mRNA) in mutant backgrounds was determined using qPCR for **(A)** *BES1*, **(B)** *BEH1*, **(C)** *BEH3*, **(D)** *BZR1*, **(E)** *BEH2*, and **(F)** *BEH4*. **(G)** Direct and indirect regulatory relationships among *BZR/BEH* family members were determined from results in **(A–F)**. A regulatory relationship was called for a gene if a greater than a 2-fold expression difference between wild-type and mutant backgrounds was measured. Both positive (arrow) and negative (bar) regulatory relationships are indicated.

We further explored the interactions among *BZR/BEH* family members that may underlie *BEH4*-dependent developmental robustness. Specifically, we hypothesized that *BEH4* acts as hub gene among *BZR/BEH* family members. Highly connected hub genes such as the well-characterized HSP90 are thought to affect robustness through their interaction with many other loci; hub perturbation results in large-scale phenotypic effects and loss of robustness (Levy and Siegal, 2008; Fu et al., 2009; Lempe et al., 2012; Lachowiec et al., 2015b). BES1 and BZR1 ChIP results (Sun et al., 2010; Yu et al., 2011) suggest that all other *BZR/BEH* family members are potential transcriptional targets of BES1 and BZR1 (Supplementary Table S1), consistent with direct or indirect regulation among family members. Further, expression of *BEH2* is up-regulated in RNAi lines in which *BES1* is targeted (Wang et al., 2013), and *BZR1* expression is reduced in *bes1-1* mutants (Jeong et al., 2015). To test our hypothesis that *BEH4* is the most highly connected gene in this gene family through its function as a transcription factor and target of other *BZR/BEH* family members, we determined the relative expression of each *BZR/BEH* family member in each single mutant background. If mean gene expression was altered more than two-fold in a given mutant background, we assumed a direct or indirect genetic interaction between the assayed and the mutated gene. Rejecting our hypothesis, we found that *BEH3* was the most highly connected gene among the *BZR/BEH* family, not *BEH4* (Figure 3). Seven connections among *BEH3* and other family members were counted, with *BEH3* affecting three family members and *BEH3* expression affected in four mutants. Two of these interactions were reciprocal, in which *BEH3* and *BEH4* affect each other, as well as *BEH3* and *BES1*. Similar to *BEH3, BEH4* affected gene expression of three family members, but only two mutants influenced *BEH4* expression. Notably, the *beh3-1* mutant showed no decrease in developmental robustness; hence, connectivity within the *BZR/BEH* family (Levy and Siegal, 2008; Lachowiec et al., 2015b) as measured by transcription, does not capture the mechanisms underlying the robustness of hypocotyl growth. This analysis did not assess interactions at the protein level through heterodimers among family members or connections of *BEH4* with genes outside the *BZR/BEH* gene family that could reveal a relationship between connectivity and robustness.

Although connectivity was not associated with phenotypic effects, gene duplicate age appeared to be associated with the number of connections among family members. *BES1* and *BZR1* are the most recently duplicated members of the family, followed by *BEH1* and *BEH2*, with *BEH3* and *BEH4* being the earliest diverged (Blanc et al., 2003). With three connections, *BZR1* and *BES1* were the least connected genes; *BEH1* and *BEH2* each showed four connections. These results are consistent with closely related transcription factors gaining regulatory complexity over time as paralogs are added (Wagner, 1996).

We and others have suggested that robustness regulators may be characterized by numerous regulatory inputs and few outputs, an architecture well suited to buffer noise (Sangster et al., 2004; Lehner et al., 2006; Levy and Siegal, 2008; Rinott et al., 2011). To further investigate the regulatory network underlying hypocotyl elongation in the dark beyond the *BZR/BEH* family, we analyzed recent DNase I-seq data of dark grown seedlings to infer regulatory connections across studied transcription factors (Sullivan et al., 2014). The promoter-proximal accessible chromatin of *BEH4* and *BEH3* each contained 25 transcription factor motifs, and 26 transcription factor motifs were found for *BEH2* and 35 for *BZR1* (Supplementary Table S2). In contrast, no transcription factor motifs were detected for *BEH1*, and only six TF motifs were found for *BES1*. We conclude that for the *BZR/BEH* gene family the number of regulatory inputs is not associated with the severity of phenotypic effects on developmental robustness or trait mean. We were unable to assess regulatory outputs because the binding motifs of individual *BZR/BEH* family members are unknown. *BES1* and *BZR1* both recognize the BZR motif, which resided in accessible, promoter-proximal chromatin of 230 genes. Although *beh4-1* most strongly affects tested phenotypes among the *BZR/BEH* mutants, neither connectivity among *BZR/BEH* family members nor the inferred transcriptional regulation of *BEH4* is consistent with the hypothesized role of *BEH4* as a hub gene relative to other *BZR/BEH* family members.

### HSP90 Likely Maintains Developmental Robustness of Dark-Grown Hypocotyls via BEH4

**FIGURE 4:**
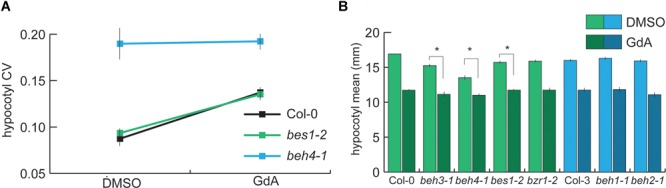
Robustness provided by HSP90 likely arises from the chaperone’s interaction with BEH4 *|* **(A)** Seedlings were grown with or without HSP90 inhibition, and hypocotyl length was measured in three replicate experiments. CV was calculated for each replicate and the standard errors of the mean for *n* = 3 are shown. BES1 is a known HSP90 client in this gene family. **(B)** Hypocotyl length mean data for the same conditions are shown. One representative replicate experiment with standard error of the mean for *n* > 20 is shown. ^∗^Significant differences in mean trait response to HSP90 inhibition are shown (*p* < 0.03, linear mixed model with genotype, treatment, and interaction effects as fixed effects and replicate as a random effect).

HSP90 function is crucial for developmental robustness of dark-grown hypocotyls and other traits (Queitsch et al., 2002; Sangster et al., 2007, 2008a,b). As HSP90 chaperones the *BZR/BEH* family member BES1 (Lachowiec et al., 2013; Shigeta et al., 2013), we hypothesized that the dominant role of *BEH4* in developmental robustness may involve HSP90. To test this hypothesis, we assessed the genetic interaction of HSP90 and *BEH4*, using the potent and highly specific inhibitor geldanamycin (GdA) to reduce HSP90 function. As previously observed, HSP90 inhibition in wild-type seedlings decreased robustness (Figure 4A). HSP90 inhibition in *bes1-2* mutant seedlings also decreased robustness, closely resembling the phenotypic effect observed in wild-type (Figure 4A). In stark contrast, *beh4-1* exhibited no change in developmental robustness upon HSP90 inhibition (*p* = 0.296, Levene’s test, *n* = 210). In fact, *BEH4* appeared to be epistatic to HSP90 in mediating developmental robustness of dark-grown hypocotyls, suggesting that HSP90 acts via BEH4.

The most obvious mechanism by which HSP90 would act via BEH4 to mediate developmental robustness is by chaperoning BEH4. The *BZR/BEH* family member BES1, but not BZR1, is an HSP90 client (Lachowiec et al., 2013; Shigeta et al., 2013). Due to the high similarity among *BZR/BEH* family members, it is likely that others are also HSP90 substrates, as client status is often shared among family members (Taipale et al., 2012; Lachowiec et al., 2015a). HSP90 inhibition typically compromises the function of its clients due to mis-folding and degradation (Taipale et al., 2010). The observed epistasis of BEH4 with HSP90 in developmental robustness (lack of response in *beh4-1* upon HSP90 inhibition) is consistent with the hypothesis that BEH4 is an HSP90 client.

To further test this hypothesis, we analyzed trait means of all single mutants of the *BZR/BEH* family members with and without HSP90 inhibition. As expected from our previous studies (Lachowiec et al., 2013), the mutant of the HSP90 client BES1, *bes1-2*, was significantly less sensitive than wild-type to HSP90 inhibition (*p* = 0.03, linear mixed effects model, *n* = 20, Figure 4B). Moreover, both *beh3-1*, and *beh4-1* were significantly less affected than wild-type (*p* = 0.01, *p* < 0.0001, respectively, linear mixed effects model, *n* = 20, Figure 4B). In contrast, *beh1-1, beh2-1*, and *bzr1-2*, whose wild-type protein is not chaperoned by HSP90 (Lachowiec et al., 2013; Shigeta et al., 2013), responded to HSP90 inhibition similarly to wild-type. These results are consistent with our hypothesis that BEH4 and possibly BEH3 are HSP90 clients.

## Discussion

Developmental robustness is hypothesized to emerge from the topology of gene networks, including the activity of redundant genes, gene connectivity, and regulatory architecture (Lachowiec et al., 2015b). Here, we trace developmental robustness of the dark-grown hypocotyl length to a specific member of the *BZR/BEH* gene family, *BEH4*. Contrary to our expectation that higher-order mutants in partially redundant genes would be necessary to decrease developmental robustness, the single *beh4-1* mutant was sufficient. A higher-order mutation in an additional family member did not further decrease developmental robustness; rather, we observed partial rescue. *BEH4*, the earliest diverged member of the *BZR/BEH* family, also showed the largest effect on the trait mean phenotype. Our observations challenge a prior theory that additional connections (here paralogs), added later in evolution, stabilize traits (Wagner, 1996). Instead, at least for this particular trait and gene family, a mutant in the earliest diverged gene has the largest effect on both developmental robustness and trait mean. Previous studies have found that loci that affect trait robustness also affect trait mean (Hall et al., 2007; Ordas et al., 2008; Sangster et al., 2008b; Jimenez-Gomez et al., 2011). This frequently observed overlap makes intuitive sense: a gene that significantly affects trait mean when disrupted will perturb the underlying stabilizing genetic network and may so decrease trait robustness (Félix and Barkoulas, 2015; Hallgrimsson et al., 2018). As stabilizing selection on genetic variants that affect both mean and variance will be far stronger than selection on variants that affect only trait variance, genes such as *BEH4* will play critical roles in maintaining phenotypic robustness.

Gene network hubs are thought to be crucial for developmental robustness, presumably due to their high number of connections with other loci. This assumption is certainly supported by several prior studies in plants, yeast and worms (Queitsch et al., 2002; Lehner et al., 2006; Sangster et al., 2007; Levy and Siegal, 2008; Rinott et al., 2011). At the scale of connectivity within the *BZR/BEH* gene family, this assumption did not hold true. We did, however, observe that the older gene duplicates, *BEH3* and *BEH4*, tended to engage in more regulatory connections than other family members, consistent with previous studies finding that number of protein interactions correlates with gene age (Eisenberg and Levanon, 2003; Kunin et al., 2004; Saeed and Deane, 2006). However, *beh3-1* did not exhibit altered developmental robustness, indicating that connectivity alone within the *BZR/BEH* family does not suffice to explain effects on developmental robustness.

The known genetic network underlying hypocotyl dark growth is complex (Oh et al., 2014), and thus far *BEH4*’s role within this network has been unknown. Our analysis of DNAse I-seq data for dark-grown seedlings revealed the putative number of TFs regulating different *BZR/BEH* family members (Supplementary Table S1). The number of potential regulatory inputs for individual family members did not correlate with the severity of the phenotypic effects in their mutants; several family members showed equal or more inputs than *BEH4*. Further study is needed to identify genome-wide targets of *BZR/BEH* family members to clarify whether and how connectivity and regulatory architecture correlates with dark-grown hypocotyl developmental robustness.

Our data are consistent with the alternative hypothesis that *BEH4*’s role in developmental robustness arises through the topology of its connections with other family members. For example, feedback loops are known to promote robustness (Hornstein and Shomron, 2006; Ebert and Sharp, 2012; Cassidy et al., 2013; Lachowiec et al., 2015b). We found that *beh4-1* decreases levels of *BEH3* and *BEH1*, both of which negatively regulate *BEH4*. Hence, loss of robustness in *beh4-1* mutants may arise through the loss of finely tuned regulation among family members. This hypothesis is supported by our observation that in the *bes1-2*;*beh4-1* double mutant, developmental robustness is partially rescued, possibly because the regulatory balance among family members is partially restored in the double mutant. This topology hypothesis is not necessarily mutually exclusive from hypotheses about degree of connectivity and regulatory architecture; all may underlie the emergence of developmental robustness.

Further work is needed to understand how the *beh4-1* mutant behaves at the molecular level and how that translates into the observed phenotypes. Expression of only the first exon is detected in *beh4-1.* Given the short hypocotyl phenotype observed in *beh4-1* and in the RNAi knock-down of *BES1/BZR1* (Yin et al., 2005; Wang et al., 2013), we suspect that this severely shortened transcript is not translated or is non-functional. Development of knock-out mutants using CRISPR-Cas9 will further clarify these findings.

The *BZR/BEH* family member BES1 is known to be a client of the developmental robustness regulator HSP90 (Lachowiec et al., 2013; Shigeta et al., 2013). HSP90 presumably governs developmental robustness by chaperoning its client proteins, which function in diverse developmental pathways (Taipale et al., 2010). HSP90 inhibition leads to destabilization and loss of function for its many clients (Xu, 1993; Taipale et al., 2012). Notably, *bes1-2* did not affect robustness, indicating that HSP90 does not regulate robustness through its client BES1. Instead, we observed that HSP90-dependent robustness of hypocotyl growth is likely due to *BEH4* function—unlike wild type, the *beh4-1* mutant showed no response to HSP90 inhibition with regard to developmental robustness. Together, this result and the significantly diminished mean response of *beh4-1* mutant to HSP90 suggest that BEH4 is also an HSP90 client. In sum, we propose that HSP90 regulates developmental robustness of dark-grown hypocotyls through the activity of BEH4, which is central for fine-tuned cross-regulation among all *BZR/BEH* family members.

## Materials and Methods

### Plant Materials and Growth Conditions

*bes1-2* (Lachowiec et al., 2013), *bzr1-2* (GABI_857E04), *beh3-1* (SALK_017577), and *beh4-1* (SAIL_750_F08) are in the Col-0 background. *beh1-1* (SAIL_40_D04) and *beh2-1* (SAIL_76_B06) are in the Col-3 background.

For hypocotyl length assays, seeds were sterilized for 10 min in 70% ethanol, 0.01% Triton X-100, followed by 5 min of 95% ethanol. After sterilization, seeds were suspended in 0.1% agarose and spotted on plates containing 0.5× Murashige Minimal Organics Medium and 0.8% bactoagar. Seeds on plates were then stratified in the dark at 4°C for 3 days and then transferred to an incubator cycling between 22°C for 16 h and 20°C for 8 h to imitate long days. Plate position was changed every 24 h to minimize position effect for light grown seedlings. Racks of plates containing dark-grown seedlings were wrapped in foil. For HSP90-inhibitor assays, 1 μM geldanamycin (Sigma) was added to the medium. An equivalent volume of the solvent DMSO was used for a control.

### Phenotyping

For estimates of hypocotyl CV, three replicates of *n* > 50 were measured. Assays of mean hypocotyl length were completed in triplicate with *n* > 15. Photos were taken of each plate, and individual hypocotyls were manually measured using NIH ImageJ1.46r.

### qPCR

Three biological replicates of sixty pooled 5-day dark grown seedlings were harvested. Tissue was frozen in liquid nitrogen and ground by hand with a pestle. RNA was extracted using the SV Total RNA Isolation kit (Promega). To remove contaminating DNA, a second DNase I treatment was completed according to the Turbo DNase protocol (Ambion). Poly-A tail cDNA was produced using LightCycler kit with oligo-dT primers (Life Technologies). Primers are listed in Supplementary Table S3, with all qPCR pairs containing a primer that spans an exon-exon junction, except for *BEH2*. In *bzr1-2, beh2-1*, and *beh4-1* mutants, primers amplified short products. The absence of the full-length transcripts in *bzr1-2* and *beh2-1* was confirmed using primers that target the full-length transcript, and for *beh4-*1, the absence of an exon 1 - exon 2 spliced transcript was confirmed using a junction spanning primer.

## Data Availability Statement

The raw data supporting the conclusions of this manuscript will be made available by the authors, without undue reservation, to any qualified researcher.

## Author Contributions

JL and CQ contributed the conception, design, and materials of the study. KS genotyped the lines. GM performed the qPCR. JL, CQ, and GM wrote the sections of the manuscript. All authors approved the submitted version.

## Conflict of Interest Statement

The authors declare that the research was conducted in the absence of any commercial or financial relationships that could be construed as a potential conflict of interest.
