# Supplementary material for: Redundancy, Feedback, and Robustness in the Arabidopsis thaliana BZR/BEH Gene Family
Source: Front Genet. 2018 Nov 13;9:523. doi: 10.3389/fgene.2018.00523 (PMC6277886; doi:10.3389/fgene.2018.00523)
Supplement: Supplementary file 1 [file Data_Sheet_1.docx]

SUPPLEMENTARY TABLES AND FIGURES

| Gene | Target of BES1^1^ | Target of BZR1^2^ |
| --- | --- | --- |
| At1g19350 BES1 | X | X |
| At1g75080 BZR1 |  | X |
| At3g50750 BEH1 | X | X |
| At4g36780 BEH2 | X | X |
| At4g18890 BEH3 |  | X |
| At1g78700 BEH4 | X |  |

**Table S1. Co-regulation of family members and response to brassinosteroids.**

^1^Yu et al 2011, ^2^Sun et al 2010. Targets of BES1 were determined with ChIP-chip using an anti-BES1 antibody on material collected from *bes1-D* 14d seedlings on plates or the BZR1 ChIP-chip (Yu *et al.* 2011). Targets of BZR1 were determined using ChIP-chip using an anti-GFP antibody on rosette tissue of transgenic BZR1-GFP fusion plants (Sun *et al.* 2010).

**Table S2. Putative transcription factors regulating members of the *BEH* family.**

| transcription factor | putative target gene |
| --- | --- |
| ABF1 | BEH2 |
| ABF3 | BEH2 |
| ABI5 | BEH2 |
| AGL9_SEP3 | BEH2 |
| AMS | BEH2 |
| AT1G01260 | BEH2 |
| AT4G17950 | BEH2 |
| AT4G29000 | BEH2 |
| ATAREB1 | BEH2 |
| AtbZIP63 | BEH2 |
| bHLH34 | BEH2 |
| BIM2 | BEH2 |
| bZIP68 | BEH2 |
| BZR1 | BEH2 |
| BZR1 | BEH2 |
| GBF1 | BEH2 |
| GBF2 | BEH2 |
| GBF3 | BEH2 |
| HY5 | BEH2 |
| ILR3 | BEH2 |
| MYC3 | BEH2 |
| MYC4 | BEH2 |
| PIL5 | BEH2 |
| POC1 | BEH2 |
| SOL1 | BEH2 |
| SPT | BEH2 |
| ZCW32 | BEH2 |
| ANL2 | BEH3 |
| APRR2 | BEH3 |
| ARR1 | BEH3 |
| ARR10 | BEH3 |
| ARR14 | BEH3 |
| ARR2 | BEH3 |
| ASIL1 | BEH3 |
| AT3G53600 | BEH3 |
| AT5G05090 | BEH3 |
| AtPHR1 | BEH3 |
| bZIP68 | BEH3 |
| CCA1 | BEH3 |
| GATA1 | BEH3 |
| GATA26 | BEH3 |
| GATA27 | BEH3 |
| GATA3 | BEH3 |
| GATA4 | BEH3 |
| GBF1 | BEH3 |
| GBF2 | BEH3 |
| GBF3 | BEH3 |
| HY5 | BEH3 |
| MYB77 | BEH3 |
| PDF2 | BEH3 |
| PHL1 | BEH3 |
| WRKY40 | BEH3 |
| AGL9_SEP3 | BEH4 |
| AT1G01260 | BEH4 |
| AT2G18300 | BEH4 |
| AT5G48560 | BEH4 |
| ATHB16 | BEH4 |
| ATHB6 | BEH4 |
| BEE2 | BEH4 |
| bHLH34 | BEH4 |
| BIM1 | BEH4 |
| BIM2 | BEH4 |
| BIM3 | BEH4 |
| bZIP68 | BEH4 |
| GBF2 | BEH4 |
| GBF3 | BEH4 |
| ILR3 | BEH4 |
| MYC3 | BEH4 |
| MYC4 | BEH4 |
| PIL5 | BEH4 |
| POC1 | BEH4 |
| RD22BP1 | BEH4 |
| SPT | BEH4 |
| TCP2 | BEH4 |
| TCP20 | BEH4 |
| UNE10 | BEH4 |
| ZCW32 | BEH4 |
| anac025 | BES1 |
| AT3G53600 | BES1 |
| AT5G51910 | BES1 |
| NAC083 | BES1 |
| TCP20 | BES1 |
| TEM2 | BES1 |
| ABI5 | BZR1 |
| AT1G01260 | BZR1 |
| AT1G64620 | BZR1 |
| AT3G53600 | BZR1 |
| AT5G51910 | BZR1 |
| AtbZIP63 | BZR1 |
| ATHB16 | BZR1 |
| ATWOX13 | BZR1 |
| AZF1 | BZR1 |
| bHLH34 | BZR1 |
| BIM2 | BZR1 |
| bZIP68 | BZR1 |
| CCA1 | BZR1 |
| CDF2 | BZR1 |
| cdf3 | BZR1 |
| DOF5.6 | BZR1 |
| GATA26 | BZR1 |
| GATA27 | BZR1 |
| GBF1 | BZR1 |
| GBF2 | BZR1 |
| GBF3 | BZR1 |
| HAT3 | BZR1 |
| HB-1 | BZR1 |
| HY5 | BZR1 |
| ILR3 | BZR1 |
| INO | BZR1 |
| MYC3 | BZR1 |
| MYC4 | BZR1 |
| PIL5 | BZR1 |
| POC1 | BZR1 |
| SPT | BZR1 |
| STZ | BZR1 |
| TCP20 | BZR1 |
| YAB5 | BZR1 |
| ZCW32 | BZR1 |

TF-binding motifs located within DNaseI-seq peaks

**Table S3. List of primers**

| Purpose | forward target | ID | Forward oligo sequence | Reverse oligo sequence |
| --- | --- | --- | --- | --- |
| qPCR | *BEH1* exon 1 & 2 | AT3G50750 | TTTGTCTTGAAGCTGGTTGGATCG | TTCTGTTGGTCGAGAACCCTTTC |
| qPCR | *BEH2* exon 2 | AT4G36780 | TATCCAACAGTGCGCCTGTGAC | AGTTTCCGCTTCGAACCACGAG |
| qPCR | *BEH3* exon 1 & 2 | AT4G18890 | TGCAATGAAGCTGGTTGGACTG | TCCATTGGTTTGCATCCCTTGC |
| qPCR | *BEH4* exon 1 & 2 | AT1G78700 | GCACTCTGTAACGAAGCTG | TGGCTGATAGGAAGAGCA |
| qPCR | *BES1* exon 1 & 2 | AT1G19350 | GGCTGGTTTAACTCAAATCAACGG | TCCGTCAGACGTCATCTTCTTCG |
| qPCR | *BZR1* exon 1 & 2 | AT1G75080 | TTGTGTTGAAGCTGGTTGGGTTG | GTAAAGGCTTGCATCCCTTGCG |
| qPCR | *UBC21* exon 2 & 3 | AT5G25760 | GACCAAGATATTCCATCCTA | GTTAAGAGGACTGTCCG |
| confirmation | *BEH2 f*ull length | AT4G36780 | CTTCAGACTCACACACACA | GGAAAGCTAAGTGGATATCATATTAC |
| confirmation | *BEH4* exon 1 | AT1G78700 | CGGAGAGCAATCGCAGCTAA | GACGATCCAACCAGCTTCGT |
| confirmation | *BZR1* full length | AT1G75080 | TGGCCGTCGCGAACCATGACTTCGGATGGAGCTAC | TCCCATTCGCGATCAACCACGAGCCTTCCC |
| genotyping | *BEH1* | AT3G50750 | TCACCTCCTTTTCACCTTTTG | GATATTAACCTAGCCGCGTCC |
| genotyping | *beh1-2* | SAIL_40_D04 | TAGCATCTGAATTTCATAACCA | GATATTAACCTAGCCGCGTCC |
| genotyping | *BEH2* | AT4G36780 | CATTGGACTCGATTCTCGAAG | GGGAGTTTTCTCGGTGAGATC |
| genotyping | *beh2-1* | SAIL_76_B06 | TAGCATCTGAATTTCATAACCA | GGGAGTTTTCTCGGTGAGATC |
| genotyping | *BEH3* | AT4G18890 | ACCTCGATCGTTGTACAAAGG | AGCCTGAGCACGTGTTAACAC |
| genotyping | *beh3-1* | SALK_017577 | ATTTTGCCGATTTCGGAAC | AGCCTGAGCACGTGTTAACAC |
| genotyping | *BEH4* | AT1G78700 | ACCACAATAGCCAAATTGCTG | TGAAATCCAAATCGCAAGATC |
| genotyping | *beh4-1* | SAIL_750_F08 | TAGCATCTGAATTTCATAACCA | TGAAATCCAAATCGCAAGATC |
| genotyping | *BES1* | AT1G19350 | CAGTCAGGACAAAAGTAAGCACTC | CCCTAAAGGTCACTTTCTCCG |
| genotyping | *bes1-2* | WiscDsLox 246D02 | AACGTCCGCAATGTGTTATTA | CCCTAAAGGTCACTTTCTCCG |
| genotyping | *BZR1* | AT1G75080 | CATCAGTCTACGTTCACACAATC | AACCAATCAAACATCAATCAATCA |
| genotyping | *bzr1-2* | GABI_857E04 | ATAATAACGCTGCGGACATCTACATTTT | AACCAATCAAACATCAATCAATCA |

**
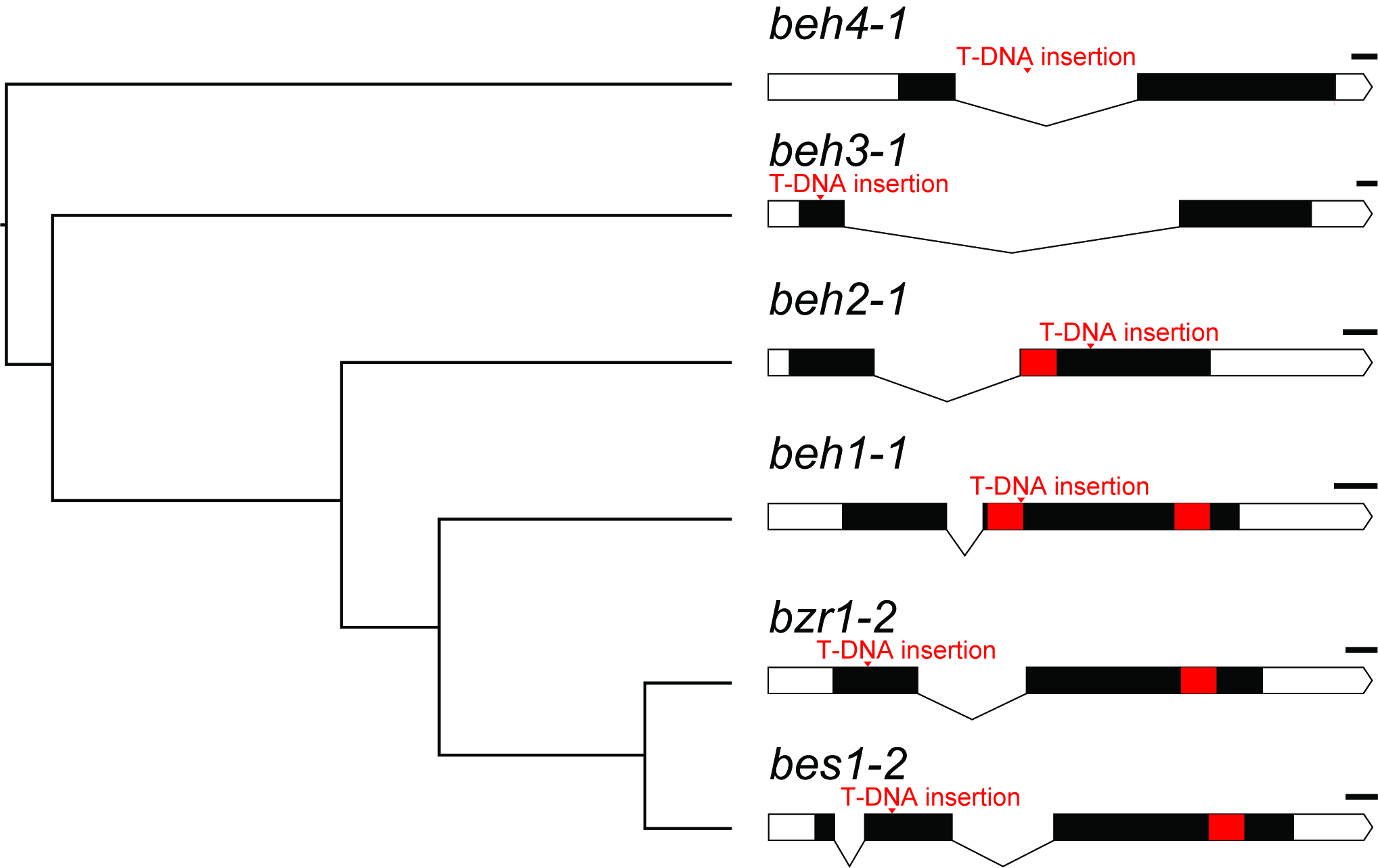
**

**Figure S1. Phylogenetic relationships and locations of T-DNA insertions and PEST domains across *BEH* family members.** Tree modified from (Lachowiec *et al.* 2013). PEST domains are marked in red.

**
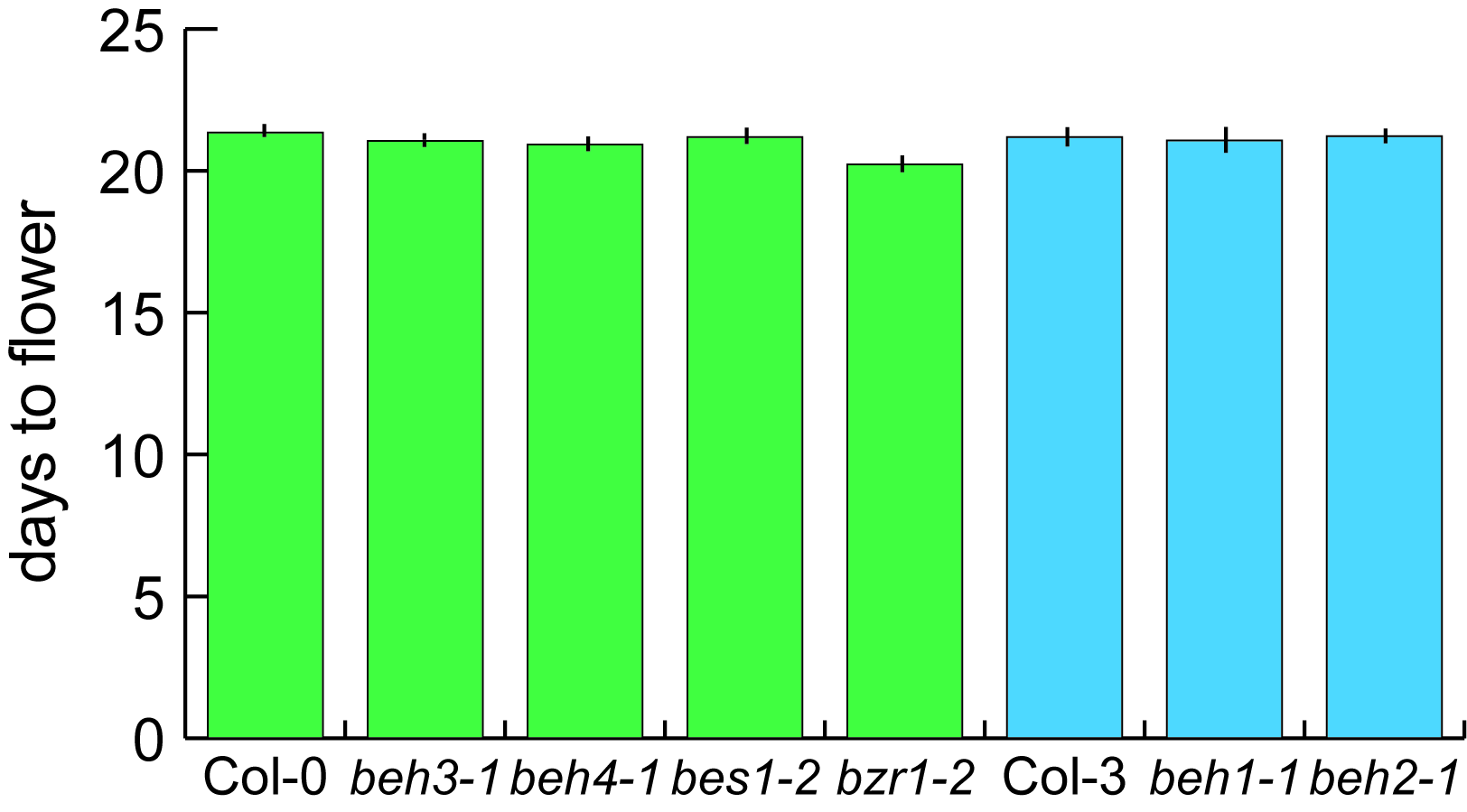
**

**Figure S2. Flowering time is not affected in single mutants in the *BEH* family.** Fifteen plants of each genotype were grown in long-day conditions at 22°. Flowering time was measured by the day of first emergence of the apical bud post-seeding.

**
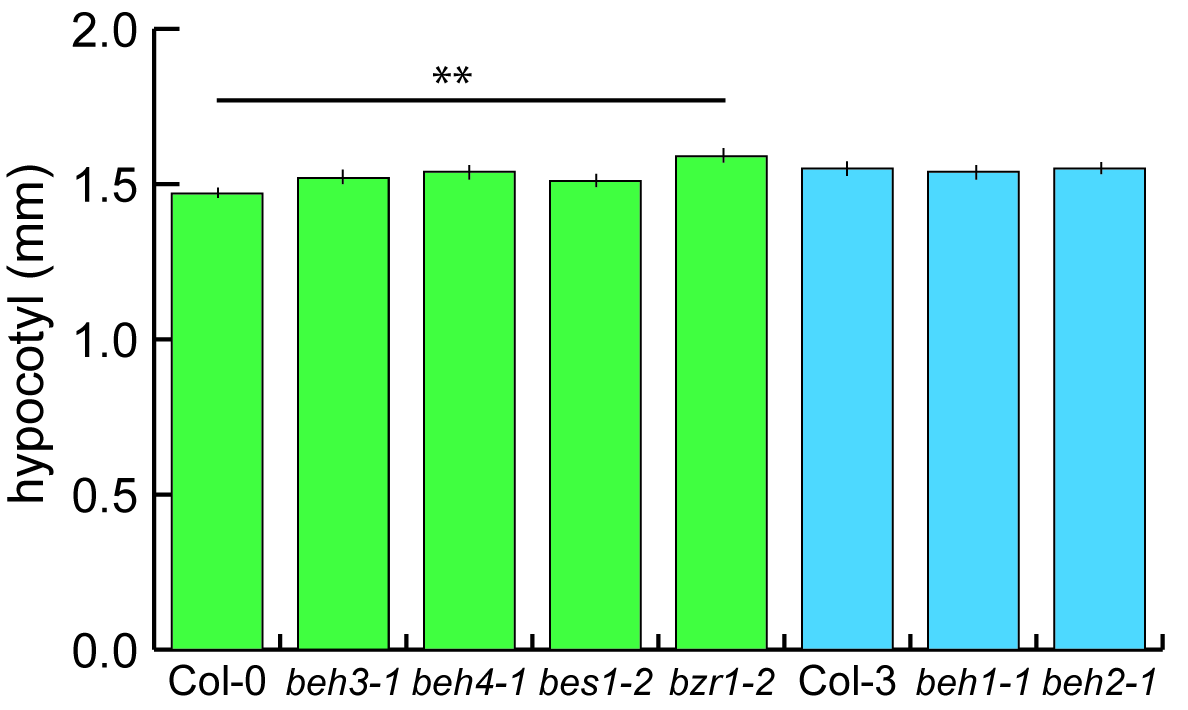
**

**Figure S3. *BZR1* negatively regulates hypocotyl growth in the light.** Seedlings were grown for seven days in long-day conditions at 22°, and hypocotyls were measured. *bzr1-2* hypocotyls were subtly but significantly longer than those of wild-type (p = 0.0087, linear mixed effects model with genotype as a fixed effect and replicate as a random effect, n = 70).
